# Chromatin loop organization of the *junb* locus in mouse dendritic cells

**DOI:** 10.1093/nar/gkt669

**Published:** 2013-08-05

**Authors:** Tamara Salem, Tiphanie Gomard, Franck Court, Gabriel Moquet-Torcy, Frédérique Brockly, Thierry Forné, Marc Piechaczyk

**Affiliations:** ^1^Equipe labellisée par la Ligue Nationale contre le Cancer, Institut de Génétique Moléculaire de Montpellier UMR 5535 CNRS, 1919 route de Mende, 34293 Montpellier cedex 5, France, ^2^Université Montpellier 2, Place Eugène Bataillon, 34095 Montpellier cedex 5, France and ^3^Université Montpellier 1, 5 Bd Henry IV, 34967 Montpellier cedex 2, France

## Abstract

The *junb* gene behaves as an immediate early gene in bacterial lipopolysaccharide (LPS)-stimulated dendritic cells (DCs), where its transient transcriptional activation is necessary for the induction of inflammatory cytokines. *junb* is a short gene and its transcriptional activation by LPS depends on the binding of NF-κB to an enhancer located just downstream of its 3′ UTR. Here, we have addressed the mechanisms underlying the transcriptional hyper-reactivity of *junb.* Using transfection and pharmacological assays to complement chromatin immunoprecipitation analyses addressing the localization of histones, polymerase II, negative elongation factor (NELF)-, DRB sensitivity-inducing factor (DSIF)- and Positive Transcription Factor b complexes, we demonstrate that *junb* is a RNA Pol II-paused gene where Pol II is loaded in the transcription start site domain but poorly active. Moreover, High salt-Recovered Sequence, chromosome conformation capture (3C)- and gene transfer experiments show that (i) *junb* is organized in a nuclear chromatin loop bringing into close spatial proximity the upstream promoter region and the downstream enhancer and (ii) this configuration permits immediate Pol II release on the *junb* body on binding of LPS-activated NF-κB to the enhancer. Thus, our work unveils a novel topological framework underlying fast *junb* transcriptional response in DCs. Moreover, it also points to a novel layer of complexity in the modes of action of NF-κB.

## INTRODUCTION

Dendritic cells (DCs) are professional antigen-presenting cells, which are key actors in the induction of adaptive and memory immunities as well as in tolerance to self-antigens [(1–4) and references therein]. Moreover, their biology has implications, not only in illnesses but also in the development of novel immunotherapies (4). In response to the capture of antigens from their environment, they undergo dramatic phenotypic and functional changes including downregulation of their phagocytic activity, acquisition of a migratory phenotype towards lymphoid organs and ability to efficiently stimulate effector lymphocytes in these organs (1–4). This maturation is associated with major alterations in the repertoire of cell surface receptors, production of soluble effectors such as proinflammatory cytokines, induction of antigen-processing and -presenting molecules, as well as with marked transcriptome reprogramming. For example, on stimulation with certain pathogen components, DCs can trigger regulatory programs involving the activation of at least 1700 genes and the repression of at least 2000 others with different kinetics for a period of 24 h (5). On their own, these data already point to a paramount role of transcription factors in DC maturation. This notion is strengthened by the fact that several percentages of the earliest induced genes are themselves transcription factors (6). The role and regulation of these transcription factors are, however, ill-defined and require further characterization for a full understanding of DC activation.

DCs are equipped with cell surface receptors that detect microbial and non-microbial products in their environment and trigger their maturation due to their signaling abilities [(2,3,7,8) and references therein]. One of the best-documented examples of DC activation is that by *Escherichia coli* lipopolysaccharide (LPS) via recognition of Toll-like receptor 4 (9,10). We have recently described an essential role for the JunB transcription factor in the transcriptional induction of the genes coding for the proinflammatory cytokines IL-6, IL-12 and TNFα (11) by LPS-activated mouse primary bone marrow-derived DCs (BMDCs). Induction of genes for such cytokines is necessary for the induction of efficient immune responses.

JunB is the protein product of the *junb* gene. It is a component of the ubiquitous AP-1 family of dimeric transcription factors that are principally made up of members of the Fos (c-Fos, Fra-1, Fra-2 and FosB) and Jun (c-Jun, JunB and JunD) multigene families (12–14). Owing to the presence of AP-1-binding sites in a large diversity of genes, AP-1 activity is crucial for all important cell decisions, including in immune cells (15). AP-1 activity is exquisitely controlled, as it is targeted by many signaling cascades and can collaborate with a number of other transcription factors to regulate the expression of its target genes. In particular, owing to the vicinity of many AP-1 (AP-1/TRE) and NF-κB (κB)-responsive DNA motifs in many promoters and the possibility of physical interaction between them, AP-1 and NF-κB co-control the transcription of various genes, including those of some cytokines [(9,16,17) and references therein]. Moreover, AP-1 and NF-κB can cross-regulate their respective expressions (11,16,17), adding a layer of complexity to their functional and physical cooperation.

In LPS-stimulated BMDCs, we have recently reported that *junb* behaves as an immediate early gene. Thus, from a basal level, the abundance of its mRNA increases rapidly, peaks by 30–90 min post-stimulation and returns to the initial level within 4 h (11). Departing from transient mRNA accumulation, JunB protein levels however increase parallely to those of mRNA but remain stable for >24 h. This is most probably due to stabilization of this intrinsically unstable protein [(18) and references therein] via mechanisms remaining to be identified. During the course of this study, we have also shown that transcriptional induction of *junb* is under the control of the NF-κB complex. Interestingly, optimal transcription of IL-6, IL-12 and TNFα genes depends on subsequent collaboration between NF-κB and JunB, which, respectively, bind to specific κb and AP-1 DNA motifs in their promoter regions (11,19–23). This revealed a regulatory pathway in which one transcription factor induces the expression of another one and subsequently collaborates with it. Such collaboration between NF-κB and AP-1 has already been documented in B cells for the induction of the CCR7 gene (17).

**Figure 1.**
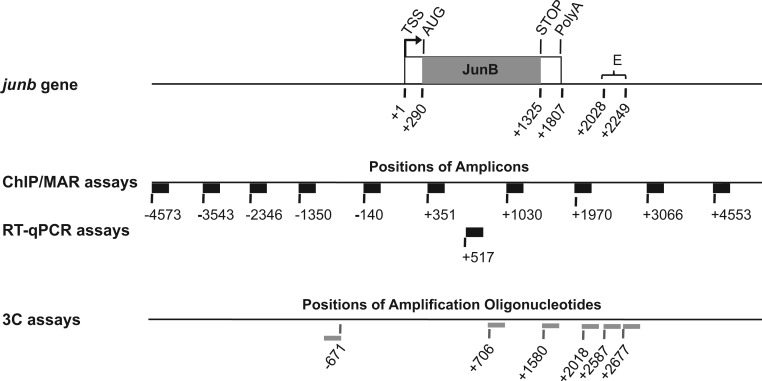
Structure of the *junb* locus. The transcribed region (1807 bp) is composed of a single exon (box with *junb* ORF in gray). The E enhancer domain is ∼200 bp long and is located 200 bp downstream of the *junb* polyadenylation site. It contains a number of binding sites for different transcription factors, including 4 κB sites of which three contribute to transcription (39–42). In the cases of ChIP-, HRS- and RT-qPCR assays, the thick black horizontal bars represent the PCR amplicons, and the numbers indicate nucleotide positions of their 5′-ends materialized by vertical bars. In the case of 3C, thin gray horizontal bars indicate the amplification oligonucleotides used. The numbers correspond to their 5′-ends, which are materialized by a vertical bar (also see Figure 7 for amplicons). Numbers are given with respect to the TSS taken as +1, as indicated in the UCSC database.

How *junb* is transcriptionally regulated is still poorly understood. *junb* is a short (1.8 kb) intron-less gene (Figure 1) expressed from low to moderate levels in most cells, including DCs. However, it behaves as an immediate early gene in response to stimuli of various sorts (growth factors, cytokines, etc), including LPS (5,6,11,17,24,25). In one hepatocytic- (26) and one neuroendocrine (27,28) cell line, *junb* has been described to be a ‘paused’ gene, though it has yet to be determined whether this also holds true in other cell types or tissues. Paused genes are genes that, when poorly active or silent, are characterized by the accumulation of RNA polymerase II (Pol II) in their transcription start site (TSS) domains but that have low, or undetectable, levels downstream (29,30). Such a distribution of Pol II on these genes is due to either transcriptional blockade or release of Pol II from the gene after transcription of short (50–100 nt) abortive non-coding RNAs (29,30). Two multimeric complexes, NELF and DSIF, are essential for the blockade (or release) of Pol II in the downstream proximal domain of the TSS (29,30), including for *junb* in hepatocytic- and neuroendocrine cells (26–28). Importantly, phosphorylations of Pol II, NELF and DSIF by CDK9, the kinase component of the Positive Transcription Factor b complex, are crucial for the relief of transcriptional inhibition (29,30). In particular, they entail the removal of nuclear elongation factor (NELF) from the TSS domain and convert DRB sensitivity-inducing factor (DSIF) from an inhibitor- to a positive elongation factor that accompanies Pol II during transcription (29,30). Furthermore, transient luciferase reporter assays, conducted in various cell backgrounds, have identified a number of DNA domains/elements located proximal to (TATA- and CAATT box regions), or more upstream of, the TSS that contribute to *junb* transcription (31–38). However, more detailed analyses have demonstrated that their contribution to *junb* transcriptional induction by various stimuli is modest (39–42). Rather, they have pointed to an essential role for an approximately 200 bp long enhancer region (E) located 200 bp downstream of the polyadenylation signal (26,39–43) (Figure 1) where several transcription factor-responsive elements have been identified (39–42), including several κB sites (39–43). Moreover, we have also reported that activation of *junb* by LPS in BMDCs correlates with binding of NF-κB in the E region (11).

The organization of the *junb* locus raises an important mechanistic question: how can the downstream enhancer transmit a transcription-activating signal to the TSS region that is located upstream of the gene body? Here, we have asked whether this might involve a chromatin loop bringing about the E and TSS regions. We have also addressed whether this loop exists prior to the recruitment of NF-κB or, on the contrary, is induced upon transcriptional activation, as has been reported for other genes by other transcription factors (see discussion). To address this issue, we have used a mouse DC cell line faithfully reproducing *junb* induction by LPS in BMDCs and a combination of RT-qPCR-, chromatin immunoprecipitation (ChIP)-, transfection and High salt-Recovered Sequence (HRS) assays as well as chromosome conformation capture (3C) experiments. Our data show that, under basal expression conditions, the *junb* locus is organized in a stable and particularly short chromatin loop that spatially juxtaposes the promoter and enhancer regions. This hitherto-unsuspected topological frame most probably explains *junb* fast transcriptional activation on LPS-induced binding of NF-κB in this locus.

## MATERIALS AND METHODS

### DC2.4 cells, stimulation by LPS and pharmacological inhibition of IKK and CDK9

DC2.4 cells are available from the ATCC and were cultured as described (44). They were stimulated with 1 µg/ml ultrapure *E. coli* LPS (InVivoGen) to exclusively stimulate Toll-like receptor 4. To inhibit IKK, 10 μg/ml BAY 11-7085 (Calbiochem) was added to cells 30 min before LPS stimulation. To inhibit cyclin-dependent kinase 9 (CDK9), cells were pre-incubated for 30 min in the presence of 100 μM of 5,6-dichloro-1 -β-D-ribofuranosylbenzimidazole (DRB) from Sigma. Both inhibitors were solubilized in dimethyl sulfoxide (DMSO). A similar volume of DMSO was added to control cells 30 min before stimulation.

### RNA preparation and RT-qPCR analyses

Total RNA from DC2.4 cells was prepared using the GenElute™ Mammalian Total RNA Miniprep kit (Sigma). After treatment with RNAse-free DNAse I (Promega), 1 µg of total RNA was used for cDNA synthesis. For this, Oligo(dT)_15_ (Promega) was used with Superscript III Reverse Transcriptase (Invitrogen), according to the supplier’s specifications. After a 10-fold dilution, 4 µl of cDNA were used for qPCR analysis using the Roche LightCycler 480 real time PCR system (Roche). The sequences of the amplification primers are presented in Table 1 (also see Figure 1). Data analyses were performed using the LightCycler software (Roche) and normalized with respect to invariant S26 mRNA levels.

**Table 1.** Oligonucleotide sequences for ChIP-, RT-qPCR- and 3C assays

Positions relative to TSS	Sequences	Orientation	Experiment
−4573	5′-CCCTGCCACTGACTATGTTTG-3′	S	ChIP, MAR (*junb*)
−4467	5′-TGGCTGGTGTCTGTGTGTATG-3′	AS	ChIP, MAR (*junb*)
−3543	5′-GCCAAGACCAAAAGCCCCAGA-3′	S	ChIP, MAR (*junb*)
−3418	5′-CCCACACCGCCTGAACTACAG-3′	AS	ChIP, MAR (*junb*)
−2346	5′-GGGCAAGATGGGAAGGAGGAC-3′	S	ChIP, MAR (*junb*)
−2153	5′-GAGGGCTCTTCAGAGAGAAGC-3′	AS	ChIP, MAR (*junb*)
−1350	5′-GCCTGTTGCCTTGGTGACGAG-3′	S	ChIP, MAR (*junb*)
−1181	5′-CAAGCGACCCTTGGGGAAGTC-3′	AS	ChIP, MAR (*junb*)
−140	5′-GCCGCTGTTTACAAGGACACG-3′	S	ChIP, MAR (*junb*)
+23	5′-CCTCAAAGTCCCCAGTGCTCG-3′	AS	ChIP, MAR (*junb*)
+351	5′-ACGACGACTCTTACGCAGCGG-3′	S	ChIP, MAR (*junb*)
+457	5′-GGACCCTTGAGACCCCGATAG-3′	AS	ChIP, MAR (*junb*)
+517	5′-CAGCTACTTTTCGGGTCAGG-3′	S	RTqPCR (*junb*)
+746	5′-ACGTGGTTCATCTTGTGCAG-3′	AS	RT-qPCR (*junb*)
−671 CONSTANT	5′-TCAGAACAAAGGTCCTGGGGA-3′	AS	3C (anchor) (*junb*)
+706	5′-CCCTGGACGACCTGCACAAGA-3′	S	3C (*junb*)
+1030	5′-AGAGGAACCGCAGACCGTACC-3′	S	ChIP, MAR (*junb*)
+1220	5′-CAGCCCCGCGTTCTCAGCCTT-3′	AS	ChIP, MAR (*junb*)
+1580	5′-TAACAGGGAGGGGAGAAGGGG-3′	S	3C (*junb*)
+1970	5′-TATCCCCTGAGTCCTGGCACC-3′	S	ChIP, MAR (*junb*)
+2018	5'-ATGTGCAAGCATGACCCCGCC-3'	S	ChIP, MAR (*junb*)
+2131	5′-CGCTGGCGTCACTGAGCTGAA-3′	AS	ChIP, MAR (*junb*)
+2587	5′-TGCTGTTGGGATCTGGGTGCC-3′	S	3C (*junb*)
+2677	5′-GTGAAGGGAACGGGCCTCAAG-3′	S	3C (*junb*)
+3066	5′-TGCTGCTTCGCCTGAACCCAC-3′	S	ChIP, MAR (*junb*)
+3259	5′-GCTCGCCTCCCTTATCCCAGA-3′	AS	ChIP, MAR (*junb*)
+4553	5′-CAGCCCCTTCAGAGAGTGGAG-3′	S	ChIP, MAR (*junb*)
+4704	5′-GGCAGTGACACCATCAAGCCC-3′	AS	ChIP, MAR (*junb*)
+15	5′-GAACATTGTAGAAGCCGCTGCTGTC-3′	S	RTqPCR (S26 mRNA)
+253	5′-AACCTTGCTATGGATGGCACAGCTC-3′	AS	RTqPCR (S26 mRNA)
+1547	5′-TTGTGTTTGTGGACGAAGTACCGAAAGGTC-3′	S	qPCR (*luc* mRNA)
+1634	5′-CCCTTCTTGGCCTTTATGAGGA TCTCTCTG-3′	AS	qPCR (*luc* mRNA)

Nucleotide numbers are relative to the *junb* TSS, as indicated in the UCSC database, and indicate the 5′ end of oligonucleotides. S and AS correspond to sense and antisense orientation with respect to the coding region of *junb*.

### Immunoblotting analyses

They were performed according to Gomard *et al.* (11) using either a monoclonal antibody to JunB (kind gift from Dr M. Yaniv, Paris), a IκBα- (Sc-371; Santa Cruz Biotechnology) or a glyceraldehyde-3-phosphate dehydrogenase (GAPDH) antiserum (Sc-25778; Santa Cruz Biotechnology). GAPDH was used as an internal invariant control in our experiments.

### Indirect immunofluorescence assays

They were performed according to Gomard *et al.* (11) using a NF-κB/p65 antiserum (Sc-372; Santa Cruz Biotechnology).

### ChIP analyses

ChIP experiments were conducted as described in Gomard *et al.* (11) using either polyclonal antibodies to (i) NF-κB/p65 (Sc-372; Santa Cruz Biotechnology), (ii) AcH3K9 (07–352; Millipore), (iii) NELF-A (Sc-23 599; Santa Cruz Biotechnology) and DSIF/hSpt5 (H-300; Sc-28678; Santa Cruz Biotechnology) or monoclonal antibodies to (i) H3 (CT, Pan, clone A3S, 05-928; Millipore), (ii) H3K4me3 (MC 315, Millipore), (iii) CDK9 (Sc-13130; Santa Cruz Biotechnology), (iv) Pol II (8WG16 MMS-126R; Covance), (v) phospho-Ser2 Pol II H5 (MMS-129R; Covance) and (vi) phospho-Ser5 Pol II (H14; MMS-134R; Covance). The H5 and H14 antibodies being IgMs, a pre-incubation of 30 min was performed with an anti-IgM antiserum (Invitrogen) to ensure their strong binding to Protein G Dynabeads (Invitrogen) during the immunoprecipitation step. Amplification primers and amplicons are presented in Table 1 and Figure 1. At least three independent chromatin immunoprecipitation (ChIP) experiments were carried out per antibody (see legends to Figures). In each experiment, enrichments of analyzed proteins on the various regions of the *junb* locus were quantified by qPCR after normalization with respect to total DNA input. Data are presented as ratios, after setting the signal on the *junb* locus E region in LPS-induced cells to 1. Each given ratio is the average of ratios obtained in multiple independent experiments. Negative controls consisted of ChIPs conducted with the GAPDH antiserum using, or not, non-stimulated cells. A control threshold value was calculated to establish the normal ratio limit. The values above this threshold pointed to significant enhancement of protein abundance. This limit was set by pooling all control values and calculating the mean of the control group and the error (mean of the control errors group). The final threshold value must be considered as Mean ± Error (dotted line in Figures).

### Plasmids and transfected constructs

**Figure 2.**
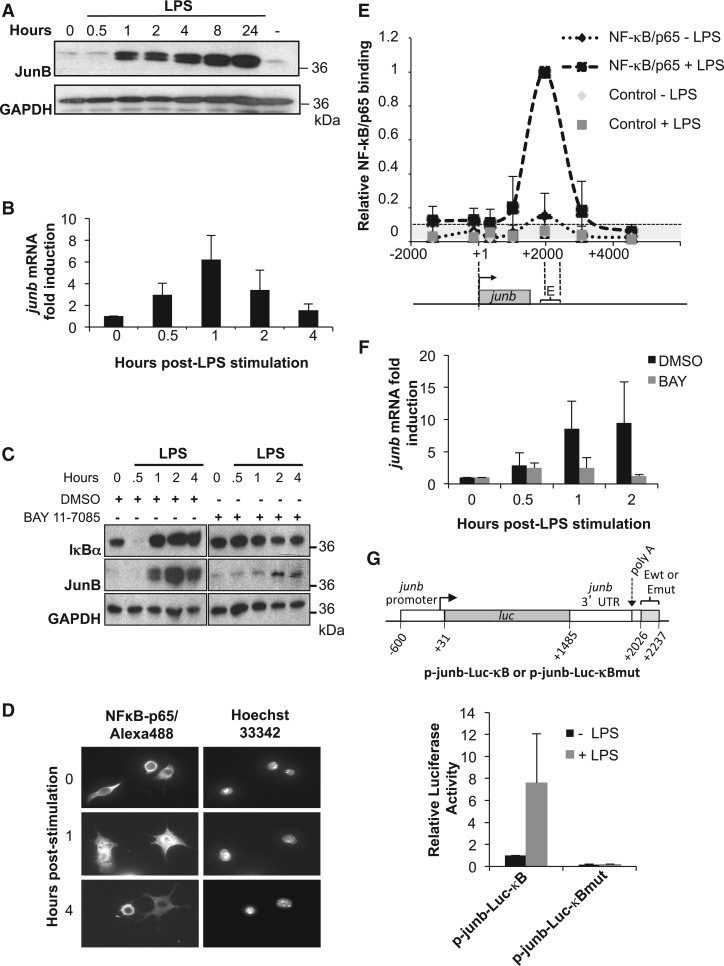
JunB induction by LPS is NF-κB dependent. (**A**) Expression of *JunB* protein. DC2.4 cells were stimulated with LPS and JunB levels were assayed by immunoblotting at different time points. A representative experiment, of 5, is shown. GAPDH was used as an internal invariant control. (**B**) Expression of junb *mRNA.* DC2.4 cells were stimulated with LPS for the indicated times and total RNA was purified and subjected to RT-qPCR analysis, as described in ‘Materials and Methods’ section. S26 mRNA was used as an invariant control. Values are the means +/− S.D of six independent experiments. (**C**) Inhibition of JunB induction by BAY 11-7085*.* DC2.4 cells were pre-treated with the IKK inhibitor BAY 11-7085 for 30 min or with DMSO as a control, and then treated with LPS for the indicated times. Total cell extracts were analyzed by immunoblotting with anti-IκBα, -JunB- and -GAPDH antisera. The results presented are representative of two independent experiments. (**D**) NF-κB/p65 nuclear translocation upon LPS stimulation*.* DC2.4 cells were left unstimulated or were stimulated with LPS for 1 and 4 h. After cell fixation, nuclei were stained with Hoescht 33342 and NF-κB/p65 was detected by indirect immunofluorescence. (**E**) LPS-induced NF-κB/p65 binding to junb. DC2.4 cells were stimulated with LPS or left untreated, and ChIP experiments were conducted as described in ‘Materials and Methods’ section to assess NF-κB/p65 binding [NF-κB/p65 (± LPS)] over the *junb* locus using qPCR quantification. Binding to the E region in LPS-stimulated cells was arbitrarily set to 1. Negative controls using an anti-GAPDH antibody [Control (± LPS)] were used to establish the significance threshold (ST), which was 0.1 (see ‘Materials and Methods’ section). The presented data are the average ± SD of five independent experiments. The *junb* locus is represented below the ChIP data for the sake of clarity. (**F**) Inhibition of junb mRNA induction by BAY 11-7085. LPS-induced cells were pre-treated for 30 min before stimulation with BAY 11-7085 or DMSO as a control and *junb* mRNA quantification was conducted as in B. The data are the means ± SD of three independent experiments. (**G**) Dependence on NF-κB sites for junb induction. DC2.4 cells were transfected for 16 h with either the p-junb-Luc-*κ*B- or the p-junb-Luc-*κ*Bmut reporter plasmid (right panel) together with a β-galactosidase reporter plasmid used as an internal standard. They were stimulated, or not, with LPS for 8 h, at which time luciferase activity was assayed (left panel). p-junb-Luc-*κ*B- or the p-junb-Luc-*κ*Bmut contain the −600/+2237 region of *junb* where *junb* ORF has been replaced by the firefly luciferase. In p-junb-Luc-*κ*Bmut, the 3 *κ*B responsive sites have been mutated to render them non-functional. The data are the means of three independent experiments ± SD.

The p-junb-Luc-*κ*B- or the p-junb-Luc-*κ*Bmut reporter plasmids that were used in Figure 2G were kind gifts of Dr M. Schorpp-Kistner (Heidelberg, Germany) and were based on the mouse *junb* gene. To construct them, the −601/+31 *junb* promoter region was fused to the firefly luciferase ORF, which itself was fused to the +1485/+2237 *junb* region that contains most of *junb* 3′UTR and its downstream region including most of the E domain (p-junb-Luc-*κ*B) or the equivalent domain where the 3 *κ*B sites crucial for NF-κB responsiveness (39) have been rendered inactive by site-directed mutagenesis (p-junb-Luc-*κ*Bmut). To construct the mP-luc-E, E-mP-luc and Emut-mP-luc plasmids used in Figure 8A, the minimal promoter (mP; −206/+31 domain with respect to the TSS) and the wild-type- (E) and κB-mutated (Emut) enhancer domains (+2026/+2237 domain with respect to the TSS) were PCR-amplified from p-junb-Luc-*κ*B- and the p-junb-Luc-*κ*Bmut, respectively, and cloned into the pGL3-Basic luciferase reporter plasmid (Invitrogen) in the positions (with respect to the luc gene) indicated in Figure 8Aa. Cloning details are available on request. Expression vectors were fully sequenced before use. To conduct the experiments presented in Figure 8B, the DNA fragments stretching from *junb* minimal promoter region (mP; starting at position −206) to the end of the E domain (till position +2237) were PCR-amplified from p-junb-Luc-*κ*B- or the p-junb-Luc-*κ*Bmut and cloned into the pCR2.1-TOPO plasmid (Invitrogen) for easier subsequent production. Before transfection experiments, the linear fragments were agarose gel-purified from the generated plasmids after cleavage at the Acc65I restriction sites present on both of their sides. To produce circular forms of these fragments, 30 µg of the purified linear fragments were ligated in a total volume of 6 ml with 0.1 units T4 DNA ligase at 25°C for 1 h. Circular forms, remnant linear DNA and polymerized fragments were then fractionated by 1% agarose gel electrophoresis and purified according to standard procedures. Two circular forms, one migrating faster than the linear fragment and the other slower, were pooled for transfection experiments. DNA fragment concentrations were assayed using the NanoDrop^R^ device. PCR amplification oligonucleotides used for clonings are presented in the Supplementary Table S1.

### Transfections and luciferase assays

In the experiments presented in Figure 2G, DC2.4 cells (5 × 10^5^ cells/point) were transfected for 16 h with Lipofectamine 2000 (Invitrogen) according to the supplier’s specifications. DC2.4 cells were then stimulated with LPS for 4 h, at which time luciferase activity was quantified using the Luciferase Reporter Assay System from Promega. In all, 800 ng of p-junb-Luc-*κ*B- or of p-junb-Luc-*κ*Bmut luciferase reporter plasmids were used per point. In all, 100 ng of a vector expressing the ß-galactosidase gene were co-transfected as an internal control of transfection. ß-galactosidase activity was measured using the X-gal test and used for normalization of data. In the experiments presented in Figure 8A, DC2.4 cells were transfected and LPS-stimulated with the mP-luc-E, E-mP-luc and Emut-mP-luc plasmids under the same conditions as in Figure 2G. In the experiments presented in Figure 8B, 500 ng of linear or circular DNA was transfected per point in DC2.4 cells together with 100 ng of a ß-galactosidase-expressing vector used as an internal control of transfection. DC2.4 cells were then LPS-stimulated as in Figure 2G or Figure 8A. Luciferase and β-galactosidase activities were then measured. Transfected DNAs were also assayed by qPCR in the same cell lysates as those used for assaying luciferase and β-galactosidase activities. This allowed us to verify that equivalent transfection efficiencies were achieved and/or that the degradation rates for the two DNA isoforms were similar. This also indicated that normalization of experiments could be achieved indifferently using the β-galactosidase assay or qPCR. The latter was achieved using oligonucleotides specific for the luciferase gene (see Supplementary Table S1). No more than 20% variations between the different transfection conditions could be observed allowing normalization of experiments using indifferently β-galactosidase or DNA assay.

### HRS assay

After lysis of DC2.4 cells, nuclei were prepared by centrifugation through a sucrose cushion (45) and HRS assays were conducted as previously described (46,47). HRSs are also called matrix attachment regions (MAR). Briefly, nuclei were treated by 2 M NaCl, and the resulting DNA ‘nuclear halos’ (owing to high salt-induced release of many proteins) were digested by the Pvu II and Sca I restriction enzymes (New England Biolabs), which allowed appropriate fragmentation of the *junb* locus (see Figure 6). The deoxyribonucleoproteic complexes precipitating on high-salt treatment and their associated genomic regions (HRS fraction) were then separated by ultrafiltration through 0.22 μm Amicon ultrafree-CL cells (Millipore) from the soluble fraction containing DNA loops (loop fraction). Genomic DNA was purified from the two fractions using phenol/chloroform extractions and ethanol precipitations. Finally, the amounts of target sequences in the HRS- versus the loop fraction were assessed by qPCR using the LightCycler 480 device as previously described (48). In each HRS assay, enrichments were defined as the ratio of the amount of target DNA in the HRS fraction versus that in the loop fraction. They were calculated and expressed relative to the ratio obtained for a negative control (NC fragment located 3543 bp upstream of the *junb* TSS, see Figure 6A), which was given the value 1 (background threshold to which all ratios were normalized). Ratios higher than 1 for which the standard deviation does not overlap with this threshold were considered as significantly enriched in the HRS fraction. The primers used were the same as those used in ChIP experiments (Table 1).

### The 3C-qPCR assays

The 3C is a proximity assay (49) in which two sequences located distantly on the DNA fiber, but spatially close in the nucleus, can be ligated together. In this assay, the amount of chimeric product formed between two sequences correlates with physical proximity *in vivo*. Therefore, specific interaction frequencies appear as local peaks in interaction profile plots where the stretched DNA fiber is presented on the abscissa. The 3C assays were conducted following the improved 3C-qPCR method described in Hagège *et al.* (50) and modified in Court *et al.* (51), except that the DdeI enzyme was used for the primary digestion and Eco RI for the secondary digestion. The original protocol is recommended for separation distances >10 kb. As the linear distances between the DNA elements we wished to interrogate in the present study were unusually small (<3 kb), we had to adapt the original quantitative 3C method (50) to obtain high resolution mapping of short range contacts while maintaining accurate measurements of interaction frequencies. To this aim, we used a frequent cutter (Dde I restriction enzyme) (see Figure 7A). This enzyme presented two advantages: (i) it is one of the rare frequent cutters whose cleavage sites are evenly distributed on the *junb* locus and (ii) it permitted us to isolate the *junb* promoter from both the gene body and the downstream region. To increase the amount of relevant 3C products, we used a previously validated primer extension approach [see (51) for details]. Briefly, eight primer extension reactions were performed (reverse primer located −624 bp relative to *junb* TSS: 5′-CCCATA AGTGGAAAAGGGAAGG-3′), pooled, purified with a QiaQuick PCR purification kit (Qiagen) and diluted in H_2_O to a concentration of 12.5 ng/μl. Each reaction was made as follows: 0.1 μM of extension primer was added to a 10 μl of reaction containing a 1× qPCR mix and 1 μl of highly concentrated ligated genomic DNA (∼200–300 ng). Primers were extended using the Hot-Start Taq *Platinium* polymerase (Invitrogen) and a LighCycler apparatus [3 min at 95°C followed by 45 cycles of (i) 1 s at 95°C, (ii) 5 s at 68°C and (iii) 15 s at 72°C]. After primer extension, amplified 3 C products were quantified by qPCR as previously described (51). Standard curves for qPCR were generated from a PCR product as follows: 150 ng of DC2.4 genomic DNA were amplified with the Expand Long Template PCR System (Roche) using the following primers: forward (located −2346 bp relative to *junb* TSS; 5′-GGGCAAGATGGGAAGGAGGAC-3′) and reverse (located +4704 relative to *junb* TSS; 5′-GGCAGTGACACCATCAAGCCC-3′). In all, 25 μl of this PCR product were digested with Dde I and ligated using T4 DNA ligase (Invitrogen) before a second digestion with Eco RI. Finally, this reaction was diluted into a solution of DC2.4 genomic DNA digested with DdeI and EcoRI (rather than in H_2_O) to obtain a final DNA concentration similar to that of the 3C reactions (12.5 ng/µl). These dilutions were used to generate standard curves for qPCR quantifications. The 3C-qPCR primer sequences are given in Table 1. Data analysis was performed using the LightCycler software (Roche), and results obtained from these experiments are included in Figure 7B.

## RESULTS

### Junb induction in DC2.4 cells

Detailed gene regulation studies require substantial amounts of cells, making them difficult or impossible to carry out in primary DCs. We therefore resorted to the model mouse cell line DC2.4. This cell line was chosen because it displays the essential properties of primary DCs (44), including in *in vivo* vaccination experiments (52), and it has already been used to conduct transcriptional studies (20).

A first important point was to validate the use of DC2.4 cells as a DC model system to investigate *junb* regulation. To this aim, we first confirmed by immunoblotting that JunB protein was induced with kinetics similar to those in LPS-stimulated BMDCs (11): from a low basal level, JunB increase became easily detectable by 1 h post-stimulation, was followed by steady accumulation for the next 7 h and remained at a high steady-state level for at least another 16 h [compare Figure 2A herein and in (11)]. Most importantly, we also verified by RT-qPCR that *junb* mRNA induction was transient. Though most often peaking by 1 h post-stimulation (Figure 2B), peaks of accumulations varied from 20 to 90 min post-stimulation with induction factors varying from 3 to 9 depending on the experiment. This variability reflected our previous observations in BMDCs (11) but had no major consequences on the interpretation of our subsequent experiments (see later in the text). Then, we controlled that NF-κB was essential for *junb* transcriptional activation using several criteria. Under basal conditions, the p65 moiety of NF-κB is retained inactive within the cytoplasm by the IκBα inhibitor. However, the latter undergoes phosphorylation by the IKK kinase and subsequent ubiquitin/proteasome-dependent degradation on signaling activation, letting NF-κB/p65 free to enter the nucleus and to activate transcription (53). We therefore first showed that *junb* induction is associated with NF-κB activation in both immunoblotting assays of transient IκBα breakdown (Figure 2C) and immunofluorescence analyses of NF-κB/p65 nuclear translocation (Figure 2D). Next, we analyzed the association of NF-κB/p65 to *junb* E region in ChIP assays covering the *junb* gene and its upstream and downstream regions (see Figure 1 for amplicon distribution) at the peak of *junb* mRNA induction. From low levels in non-stimulated cells, inducible binding in the E domain correlated with *junb* induction (Figure 2E). Then, we showed that inhibition of IKK by a selective pharmacological inhibitor (BAY 11-7085) (54) prevented, not only IκBα degradation (Figure 2C) but also *junb* mRNA (Figure 2F) and protein (Figure 2C) accumulations. This functionally implicated the NF-κB pathway in *junb* induction by LPS. This pharmacological approach, which was formerly validated in BMDCs (11), was preferred over transfection of dominant negative IKK or IκBα variants to block the NF-κB pathway, as the low transfection efficiency of DC2.4 cells precluded a significant decrease of endogenous *junb* mRNA. Finally, we verified that NF-κB-dependent induction of *junb* required the E enhancer. To this aim, we resorted to transient transfection assays using a reporter plasmid where the firefly luciferase gene was placed under the control of both the *junb* promoter and the E enhancer-containing region (see ‘Materials and Methods’ section for more details) that was either wild-type (p-junb-Luc-*κ*B) or mutated (p-junb-Luc-*κ*Bmut) on the κB sites that are essential for induction by NF-κB (39–43). On LPS stimulation, the wild-type construct, but not its mutated counterpart, was transcriptionally induced (Figure 2G), indicating that in DCs, as in other cell types (26,39–43), *junb* induction is principally under the transcriptional control of the E region. Taken together, our data indicate that DC2.4 cells constitute a reliable system to study *junb* induction by LPS.

### Histone distribution on the junb locus in DCs

**Figure 3.**
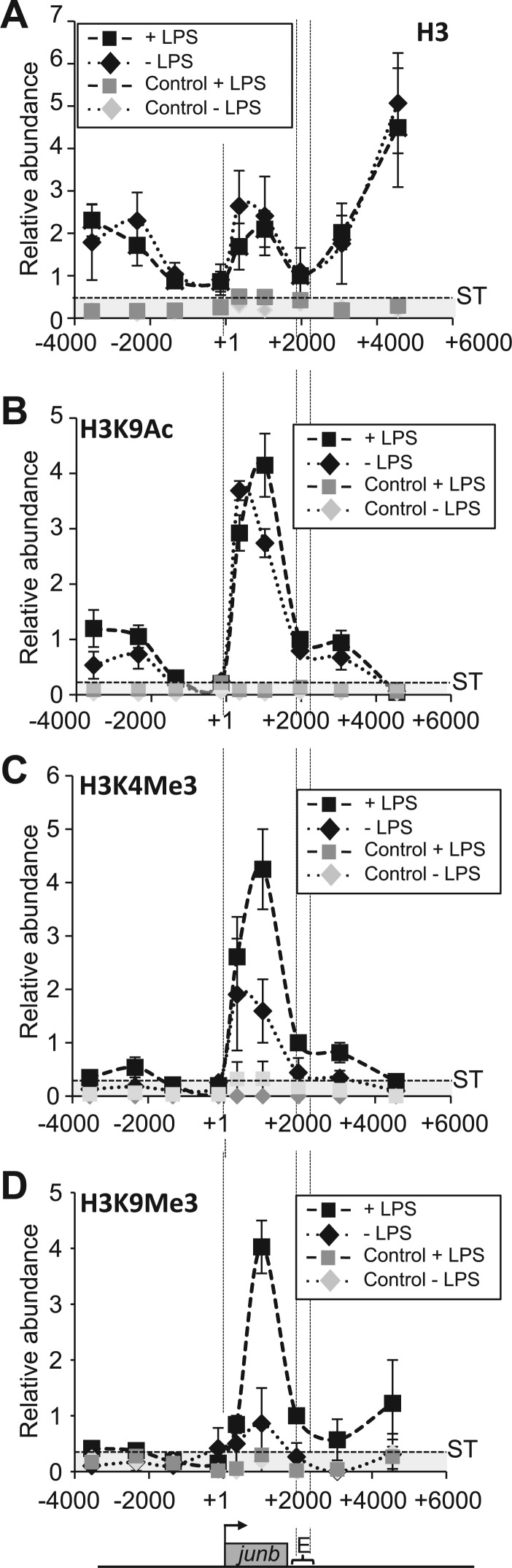
Histone distribution and modification on the *junb* locus. (**A**) Histone distribution over the junb locus*.* DC2.4 cells stimulated (+ LPS), or not (− LPS), with LPS and ChIPs were performed with a specific anti-H3 antibody. Relative abundances at various places on the locus were assayed by qPCR. They were normalized with respect to DNA inputs and presented as a ratio to LPS-induced cells with the value on the E domain arbitrarily set to 1. Negative controls using an anti-GAPDH antibody (Control ± LPS) were used to establish the significance threshold (ST), which was 0.5. The presented data correspond to values obtained at the peak of *junb* stimulation and are the average of two experiments ± SD. Points are linked with dotted lines to make the figure clearer. (**B–D**) H3 modifications. The same experiments as in (A) were carried out, except that ChIP experiments were conducted with specific anti-H3K4Me3 (B), H3K9Ac (C) and H3K9Me3 (D) antisera. ST were 0.007, 0.2 and 0.6 in B, C and D, respectively.

As a first step to our transcriptional studies, we investigated histone distribution on the *junb* locus, starting ∼3.5 kb upstream and ending ∼4.5 kb downstream of the TSS. Owing to the aforementioned variability in the position of the peak of transcriptional activity on the *junb* gene after LPS stimulation, we chose to take into consideration only two conditions: basal *junb* gene activity in non-stimulated cells and maximal transcription activity after LPS addition. This was assayed by RT-qPCR of *junb* mRNA in LPS-stimulated DCs in kinetic experiments in the same samples as those for ChIPs. In non-stimulated cells, H3 (Figure 3A) and H4 (not shown) ChIPs revealed two regions of lower nucleosome density whether *junb* was induced: one contained the TSS and ∼1000 bp just upstream and the other was centered on position +2000, i.e. contained E. Such regions [often called nucleosome-free regions (NFR)] most often mark regulatory regions of transcription-competent/active genes, revealing recruitment sites of transcription factors/cofactors/machineries (55–57) but are absent from non-expressed genes. Finding an NFR by the TSS was not surprising because we found *junb* to be a paused gene (i.e. loaded with Pol II at the TSS) in DCs (see later in the text). However, it was interesting to note the presence of an NFR containing the E domain in non-stimulated DC2.4 cells (i.e. with no NF-κB/p65 bound) and not just under conditions of stimulation by LPS (i.e. in the presence of bound NF-κB/p65) (also see later in the text).

Next, we studied histone marks associated with either active (H3K9Ac, H3K4Me3) or inactive (H3K9Me3) transcription (58,59). H3K9Ac (Figure 3B) and H3K4Me3 (Figure 3C) were principally detected on the *junb* gene body before stimulation, which was consistent with basal transcriptional activity, with a stronger accumulation at the beginning of the gene. On stimulation by LPS, H3K9Ac and H3K4Me3, both increased on the *junb* coding region, which were two observations consistent with increased transcriptional activity. Interestingly, H3K9Me3 (Figures 3D) was present at low levels in unstimulated DCs but accumulated on the *junb* gene body at the peak of transcription, though its increase was slightly delayed compared with that of H3K4Ac (Supplementary Data 1), consistently with its putative repressive role. Taken with the analysis of the other histone marks, this was suggestive of a scenario whereby, on LPS stimulation, the *junb* gene was subjected to histone modifications with antagonistic effects to ensure only transient expression.

Thus, nucleosome distribution was consistent with the idea that the main determinants of *junb* transcriptional regulation are confined to two regions in DCs: (i) the E region and (ii) the TSS + ∼1000 bp upstream of it. Moreover, the changes in both transcription-active and -inactive histone marks were suggestive of subtle dynamic chromatin changes on the *junb* locus.

### Pol II distribution on the junb locus in DCs

**Figure 4.**
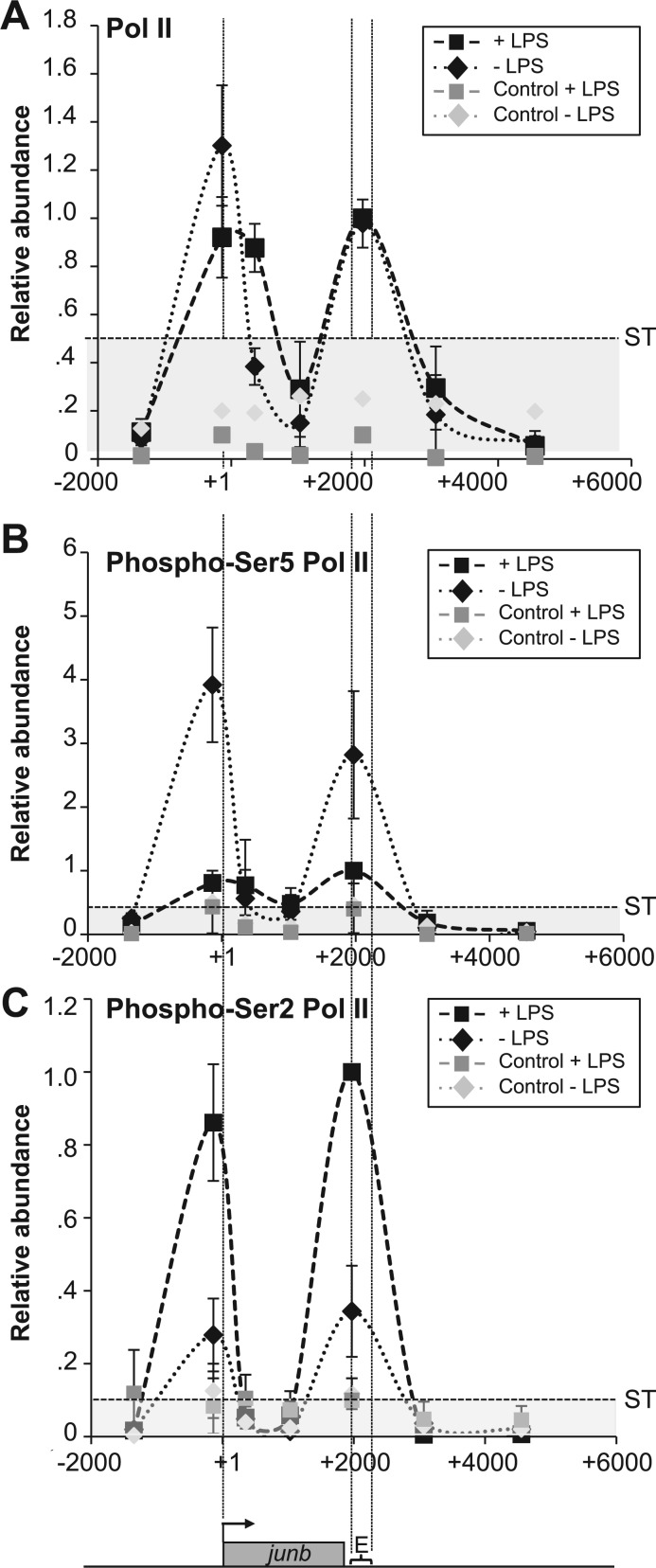
Pol II distribution and modifications on the *junb* locus. (**A**) Distribution of Pol II. DC2.4 cells were stimulated, or not, by LPS and ChIP experiments were conducted with a specific anti-Pol II antiserum. ChIP procedures, controls and quantification of relative abundances were the same as in Figure 3. The presented values are those obtained at the peak of LPS induction. The data are the means of three independent experiments ± SD. ST was 0.45. (**B**) Distribution of phospho-Ser5 Pol II. Experiments were conducted as in A with a specific anti-phospho-Ser5 Pol II antibody. ST was 0.4 (**C**) Distribution of phospho-Ser2 Pol II. Experiments were conducted as in A with a specific anti-phospho-Ser2 Pol II antibody. ST was 0.1.

We then analyzed Pol II distribution on the *junb* locus in the −1350/+4500 region. In non-stimulated DC2.4 cells, ChIPs reproducibly revealed two similar peaks of Pol II accumulation with hardly any detectable polymerase in between (Figure 4A). One contained the TSS and the other the E region. Consistently with higher transcriptional activity, Pol II abundance increased on the gene body on LPS stimulation (see amplicon 351, Figure 1), though bimodal distribution of Pol II was essentially conserved (Figure 4A). Not finding more Pol II at all tested positions on the gene body was puzzling, at first sight but is most probably explained by a combination of facts: (i) the Pol II polymerization rate (≥1200 nt/min) is extremely fast as compared with the short size of the *junb* gene, (ii) the transcription peak is transient (most probably in the few-minutes range) and (iii) cells cannot be perfectly synchronized.

We also investigated Pol II C-terminal domain (CTD) phosphorylation occurring on serines 2 and 5 of its 52 heptad repeats, as they are known to regulate Pol II activity (60,61). In keeping with the idea that Ser5 phosphorylation is principally associated with ‘paused’ and/or transcription-initiating forms of Pol II (60,61), much higher levels of phospho-Ser5 were observed in non-stimulated cells than at the peak of transcriptional activation by LPS (Figure 4B). It was however surprising to find phospho-Ser5, not only by the TSS but also in the E domain, as Ser5 is usually poorly or not phosphorylated in the 3′ regions of genes, even when they are actively transcribed (62). Phospho-Ser2, which is principally associated with the ‘elongating’ form of Pol II (60–62), was present at low level in non-stimulated DCs consistently with basal transcriptional activity. In contrast, its abundance increased dramatically when *junb* transcription was maximal after LPS stimulation (Figure 4C), possibly due to higher levels of CDK9 on *junb* (see later in the text). It is worth noting that, similarly to phospho-Ser5, phospho-Ser2 appeared as two peaks centered on the TSS and E domains when detectable. It was however intriguing to find it associated with *junb* TSS, as this modification is usually not found at the beginning of genes but rather on their body and/or at their end (60,61).

**Figure 5.**
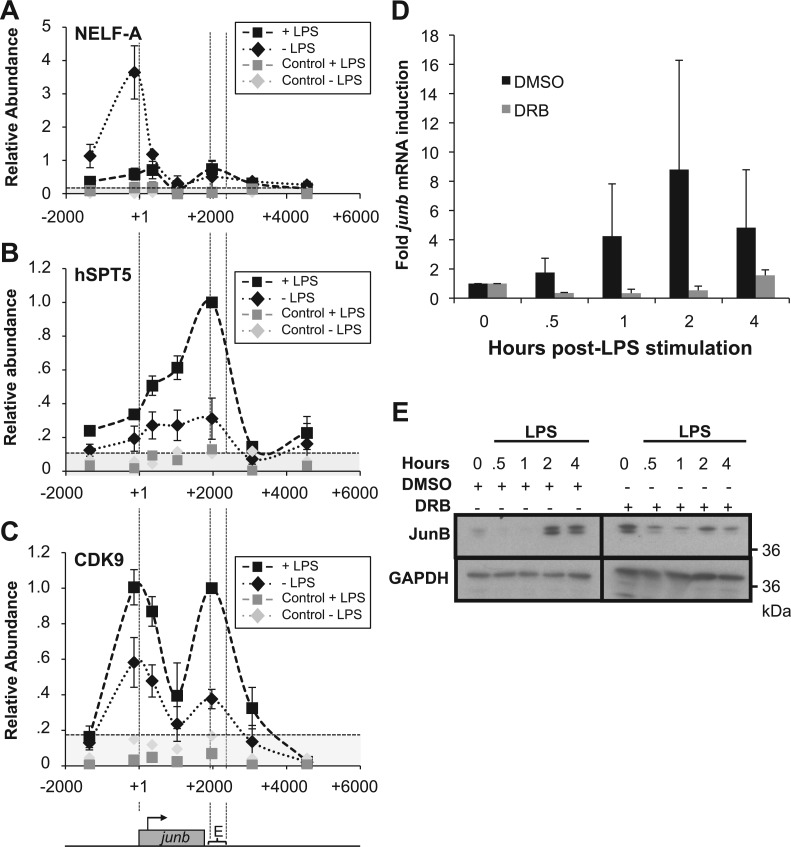
NELF and pTEF-b on *junb*. (**A–C**) Distribution of NELF-A, hspt5 and CDK9. Experiments were conducted as in Figure 4A with antibodies specific for NELF-A (C), Hspt5 (**D**) and CDK9 (**E**). ST were 0.2, 0.1 and 0.16, respectively. (D and E) Dependence on CDK9. DC.4 cells were pre-treated with DRB (or DMSO for control cells) for 30 min and stimulated, or not, with LPS. The abundance of *junb* RNA and protein were assayed by RT-qPCR (D) and immunoblotting (E), respectively, as in Figure 2A and B. Luminograms for DRB-treated cells were exposed for longer periods to make clearer the absence of JunB induction, explaining that time 0 is more intense in the right panel.

Next, owing to their implication in the control of Pol II pausing, we assessed the presence of NELF and DSIF complexes in ChIPs using antibodies directed to their NELF-A and hspt5 components, respectively. In non-stimulated DCs, NELF was essentially found on the TSS and decreased to low level on transcriptional activation of *junb* by LPS (Figure 5A). DSIF was found associated with *junb* from the TSS to the E domain in non-stimulated cells (Figure 5B). On stimulation by LPS, its abundance increased on the *junb* gene body with maximal accumulation by E (Figure 5B), which was consistent with the idea that, from an inhibitor form, it can be turned into an activator accompanying Pol II till the end of the *junb* gene (29).

Phosphorylation of Ser2 and relief of transcriptional blockade of paused genes have largely been attributed to the CDK9 component of positive transcription factor b (P-TEFb), which also phosphorylates NELF and DSIF (29,60–62). In keeping with this idea, CDK9 was found on *junb* in non-stimulated cells and higher levels were detected at the peak of transcriptional activation by LPS (Figure 5C) with a bimodal distribution reminiscent of that of Pol II in both cases (Figure 4). Moreover, the inhibition of CDK9 by its specific pharmacological inhibitor, 5,6-dichloro-1-β-DRB, abolished induction of both *junb* mRNA (Figure 5D) and JunB protein (Figure 5E) on stimulation by LPS.

Thus, taken together, our data suggest that (i) *junb* is a ‘paused’ gene in non-stimulated DCs with accumulation of NELF, DSIF and Pol II principally phosphorylated on CTD Ser5 in the TSS region, consistent with basal transcription and (ii) pausing is relieved on transcriptional activation, which is associated with more efficient recruitment of CDK9, presumably phosphorylating CTD Ser2 (and maybe other substrates), as described in liver cells stimulated by IL-6 (26). The finding of Pol II (including in its phosphorylated forms) and CDK9 accumulating in both TSS- and E-containing domains, taken with the presence of NFRs on either side of the gene, was however intriguing and raised the non-exclusive possibilities of complex transcriptional patterns or of specific chromatin organization at the *junb* locus. It was also possible that the bimodal distribution of Pol II before and after LPS stimulation may have different reasons (see ‘Discussion’ section).

### Presence of HRS regions on either side of the *junb* gene

To explain the bimodal distribution of Pol II on the *junb* gene, we first assessed the possibility of a complex transcriptional pattern at this locus. Sensitive RT-qPCR assays using first random hexanucleotides for priming reverse transcription and then specific oligonucleotides for amplification of small amplicons, did not support the idea of sense transcription downstream of E and antisense transcription from E towards the TSS. They did not support sustained antisense transcription in the proximal upstream region of the TSS either (not shown). We however do not exclude that minor transcription of small labile RNAs could occur at *junb*, as has been observed on certain genes (63).

Our ChIP protocol involved a fixation step before nuclei sonication (see ‘Materials and Methods’ section) possibly cross-linking chromatin elements distantly located on the DNA fiber but spatially close. The detection of Pol II and CDK9 ChIP signals in the E region (Figures 4A, 5A and B) might therefore be a consequence of tight chromatin loop organization of the *junb* locus bringing into proximity the E region and the TSS domain to permit reactive transcriptional activation on LPS activation. In other words, antibodies directed to Pol II or CDK9 could have immunoprecipitated the TSS- as well as the E domain, even though recruitment of these two proteins depends on determinants located in the TSS region. To assess this possibility, we first resorted to the so-called HRS assay.

**Figure 6.**
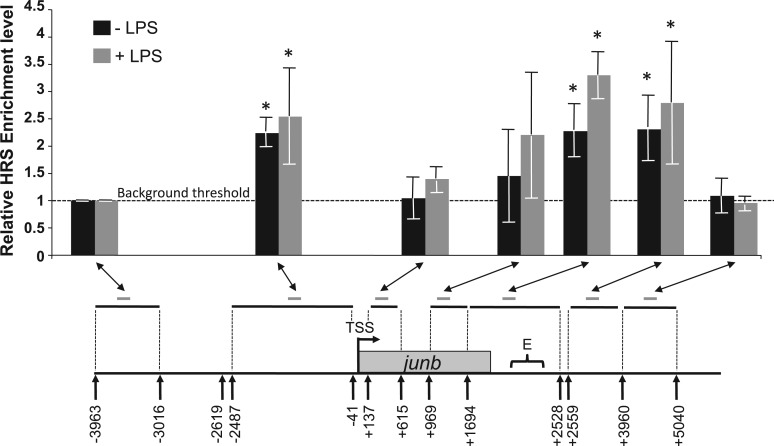
HSR at the *junb* locus. DC2.4 cells were stimulated (gray bars), or not (black bars) with LPS and HRS assays were conducted as described in ‘Materials and Methods’ section. Pvu II and Sca I restriction enzymes were used to fragment the locus. They were the only restriction enzymes we found functional in this assay. Their recognition sites are indicated by arrows. Restriction fragments analyzed in the genomic HRS assay are indicated by horizontal black lines whereas the amplicons used to quantify them (see Table 1) are indicated by short gray lines. The presented data are the means of four independent experiments ± SD. The histogram shows the relative enrichment levels of various regions of the *junb* locus in the HRS assays relative to the enrichment level of a negative control (−3963/−3016 fragment) arbitrarily set to 1. The enrichment level of this negative control is considered to be the background threshold of our experiments. Therefore, enrichment levels >1 and for which the standard deviation does not overlap with this value can be considered as significantly enriched in the HRS fraction. These regions are indicated by stars on the graph. +LPS corresponds to data obtained at the time of maximum transcription activity.

Work by two of us previously showed that the genomic regions at the basis of chromatin loops can often be evidenced using the so-called HRS assay. This assay allows trapping of such sequences into deoxyribonucleoproteic complexes that precipitate on high-salt treatment of cell nuclei (46,47). They can, then, be easily separated (by simple ultrafiltration) from DNA loops (which have been freed of most of their associated proteins by the high salt treatment), after these loops have been cleaved with appropriate restriction enzymes. Following separation, the ratios of sequences of interest in the HRS- versus the loop fractions are quantified by qPCR, and enrichment levels are calculated relative to a negative control sequence that does not organize chromatin loops (this negative control is given an arbitrary value of 1). Only target sequences for which the enrichment levels are higher than 1 (and for which the standard deviation does not overlap with this value) are considered as HRSs (for more details, see ‘Materials and Methods’ section and Figure 6) (46). The data presented in Figure 6, which are based on a fragmentation of the *junb* locus by the Sca I and Pvu II restriction enzymes, point to the existence of two HRSs in the *junb* locus. One is located upstream of the TSS (between −2487 and −41 relative to the TSS) and contains the upstream *junb* NFR. The other one is located downstream of the *junb* transcribed region (between +1694 and +3960 relative to the TSS) and contains the E region. Unfortunately, the lack of other suitable restriction enzyme sites (i.e. accessible to their cognate enzymes in the HRS assay) did not allow us to narrow down with more precision the limits of these two HRSs (not shown). It is worth noting that we did not detect any significant change in *junb* HRSs between unstimulated and LPS-stimulated DC2.4 cells.

In conclusion, independently of LPS stimulation, the *junb* gene is flanked by two HRSs that are suggestive of two attachment sites of this locus to proteinacious intranuclear structures. Although not directly demonstrating that these two domains interact physically *in vivo*, these data are consistent with the idea of a loop organization of the *junb* locus.

### Loop conformation of the *junb* locus

**Figure 7.**
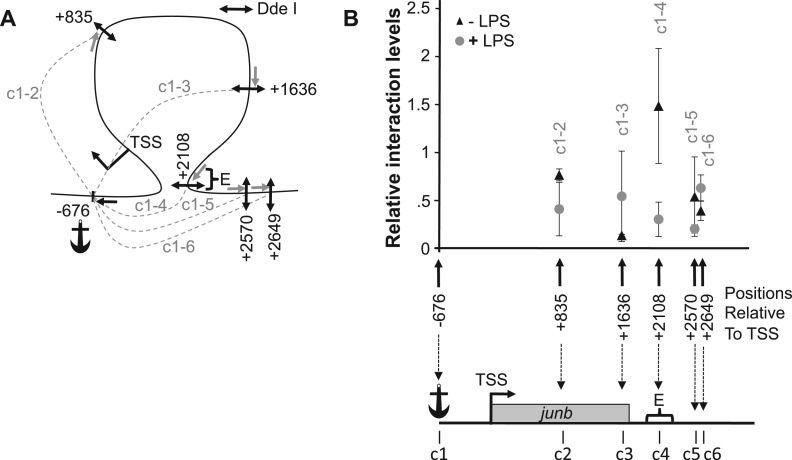
The 3C analysis of the *junb* locus. DC2.4 cells were treated (+LPS), or not (+LPS), by LPS and subjected to quantitative 3C analysis as described in ‘Materials and Methods’ section. (**A**) Map of the investigated interactions. The frequent cutter Dde I restriction enzyme was used to fractionate the *junb* locus, as we found it to be the only restriction enzyme that permits sufficiently resolutive analysis of this short locus. The positions of the Dde I sites (double arrows) used in this 3C analysis are indicated c1 to c6. c1 was taken as the anchor from which possible interactions with other region of the *junb* locus were assessed. Their locations are indicated relative to the *junb* TSS taken as +1/−1. The possible interactions that have been tested in this 3C experiments are indicated by dashed lines, which are labeled c1-c2 to c1-c6. The anchor amplification oligonucleotide used in 3C qPCR is indicated by a black simple arrow, whereas the other primers are indicated by gray ones. (**B**) Quantification of 3C analyses of the junb locus. The data represent the relative interaction frequencies between the anchor region (c1) containing the *junb* TSS and the various other tested sites (c2 to c6) of the *junb* locus. Relative interaction frequencies were determined by qPCR relative to standard curves as previously described (50,51). Data points represent the mean of four independent experiments ± SD. In the absence of LPS (black triangles), a strong interaction between the *junb* promoter and the downstream E element was observed (local peak for the c1-4 chimera). However, 1 h after LPS addition (gray circles), no specific interactions could be found.

To formally test that the *junb* locus can be organized in a chromatin loop bringing together the TSS and E domains in DC2.4 cells, we resorted to the 3C assay, a technique that was designed to assess spatial proximity of DNA elements in their native chromatin context (64). To allow precise quantification of conformation changes, we used an improved quantitative 3C assay (3C-qPCR) (50), which was adapted to the study of short-range interactions (see ‘Materials and Methods’ section and Figure 7A for more information). In these experiments, we used the Dde I restriction enzyme to fractionate the *junb* locus, as we found it to be the only restriction enzyme that permits sufficiently resolutive analysis of this short locus. Moreover, we took the Dde I site at position −676 (c1) as an anchor to test spatial proximity with other Dde I sites scattered on the *junb* locus (indicated c2 to c6 in Figure 7A). The main reasons for this choice was that c1 falls within the upstream *junb* NFR, i.e. a region that putatively binds factors essential for transcription initiation and that is prone to receive ‘signals’ from the *junb* enhancer domain. Relative interaction frequencies with the other Dde I sites of the *junb* locus (indicated by doted lines and referenced c1-2 to c1-6 in Figure 7A) were then qPCR-assayed. In non-stimulated DCs (black triangles in Figure 7B), the anchor site (c1) was found to interact more frequently with site c4 located in the E region (site 4 at position +2108) than with any of the other site tested (compare c1-4 interaction frequency with c1-2, c1-3, c1-5 and c1-6 ones in Figure 7B). Interestingly, this loop conformation was disrupted when *junb* transcription was activated after LPS stimulation (grey circles in Figure 7B).

In agreement with the HRS assay data of Figure 6, these 3C-qPCR data demonstrate that in the non-stimulated DCs, the *junb* locus is organized into a small chromatin loop that brings the enhancer and the proximal promoter regions into close physical proximity. This structure is, at least partially, relaxed after transcriptional activation by LPS. However, the HRS assays suggests that, in LPS-stimulated cells, both the 5′ and 3′ regions of the *junb* gene probably remain in contact with still-to-be-identified nuclear structures.

### Proximity of the TSS and enhancer regions of *junb* favors transcriptional activation by LPS

The simplest explanation for spatial proximity of TSS- and E domains in non-stimulated DCs is a chromatin conformation favoring transcriptional reactivity of *junb*, consistent with it being an immediate early gene. To test this possibility, we proceeded in two steps, taking into consideration that transient transfection of reporter plasmids, such as those presented in Figure 2G allows a recapitulation of NF-κB-dependent induction of *junb* by LPS, possibly because plasmids are circular structures favoring encounters between the promoter and the enhancer regions of *junb*.

**Figure 8.**
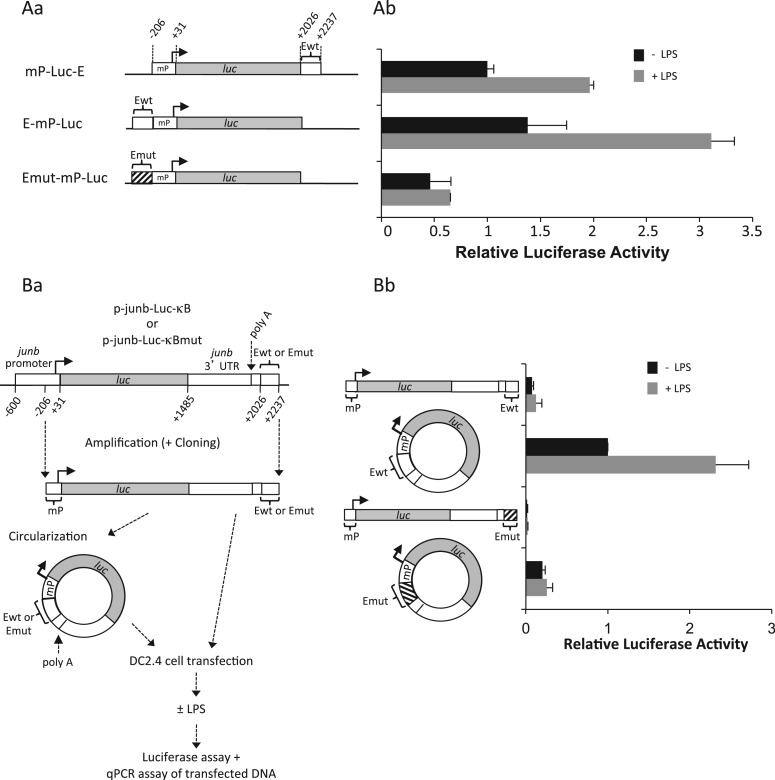
Stronger transcriptional activity in response to LPS stimulation upon forced proximity of *junb* promoter and enhancer regions. (**A**) Transfection of mP-luc-E, E-mP-luc and Emut-mP-luc plasmids in DC2.4 cells. *junb* minimal promoter (mP), wild-type E domain and E-domain mutated on the NF-κB-responsive sites were cloned upstream or downstream of the luciferase gene (*luc*) of the pGL3 reporter plasmid as indicated in Aa. mP corresponds to positions −206/+31 in mouse *junb* and E to positions +2022/+2237. DC2.4 was transfected, stimulated and processed for luciferase assay as in Figure 2G. Plasmids were cleaved with the Ase I restriction enzyme that cuts on both sides of the mP-luc-E, E-mP-luc and Emut-mP-luc fragments to avoid bias linked to the circular nature of plasmids. The presented data are the results of three independent experiments (Ab). (**B**) Transfection of linear and circular fragments bearing chimeric luc/junb genes*.* DNA fragments spanning the minimal *junb* promoter (starting at position −206) to the end of the E domain (position 2237) were purified from the p-junb-Luc-*κ*B- and the p-junb-Luc-*κ*Bmut reporter plasmids used in Figure 2G. They were then circularized using the T4 DNA ligase as described in ‘Materials and Methods’ section. DC2.4 cells were then parallely transfected with the linear and circular isoforms of these fragments and LPS-stimulated as described in Ba before assays of both luciferase activity and luciferase DNA in cell lysates. The latter DNA assays showed comparable amounts of the DNA isoforms at the end of the experiments. The results of luciferase assay after normalization of data are presented in Bb. They correspond to four independent experiments. Details of experimental procedures are given in ‘Materials and Methods’ section.

First, taking into consideration that a minimal promoter of ∼200 bp around the TSS has already been defined (39,40), we constructed expression vectors in which (ii) this promoter was cloned upstream of the luciferase (*luc*) reporter gene whose size is approximately that of *junb* and (ii) the E domain was cloned either downstream of *luc* or just upstream of the promoter (Figure 8A). These plasmids were then transfected, in linearized form, in the DC2.4 cells, which were subsequently stimulated by LPS, or not, prior luciferase activity assay. The data presented in Figure 8Ab show that LPS stimulation was stronger when the E domain was placed just upstream of the luciferase gene. Moreover, LPS stimulation was verified to be dependent on NF-κB as mutagenesis of the responsive κb sites within the enhancer placed upstream of the TSS abolished LPS induction (Figure 8Ab).

Second, the fragments −206 to +2237 (i.e. containing the minimal promoter down to the E enhancer region) of the p-junb-Luc-*κ*B- or the p-junb-Luc-*κ*Bmut reporter plasmids used in Figure 2G were transfected into DC2.4 cells either in a linear form or after circularization by T4 DNA ligase (Figure 8Ba). In the latter situation, ligation forced proximity between E and the minimal promoter, whereas, in the former, functional interactions between the two elements should be less frequent owing to the flexibility of the linear molecules. Then, luciferase activity was measured after, or without, stimulation by LPS to compare transcriptional activation in situations mimicking linear and loop conformations of the *junb* locus. Importantly, qPCR quantifications at the time of luciferase activity assay showed similar amounts of linear and circular DNA molecules. This excluded the possibility that one was transfected or degraded differently from the other. The data presented in Figure 8Bb show that forced promoter-enhancer proximity in the circular p-junb-Luc-*κ*B fragment led to stronger LPS stimulation than when transfecting its linear isoform. This stimulation was shown to be NF-κB-dependent, as no LPS induction was found when the κb sites were mutated. It is worth noting that lower basal transcription was observed in linear p-junb-Luc-*κ*B-transfected DC2.4 cells that were not stimulated by LPS. This observation is consistent with the fact that we detected (i) less basal transcription in the case of E-mP-luc as compared with Emut-mP-luc in the experiments presented in Figure 8Ab and (ii) less basal transcription in the case of p-junb-Luc-*κ*B than in that of p-junb-Luc-*κ*Bmut in the experiments presented in Figure 2G (see ‘Discussion’ section).

Thus, forced proximity of the *junb* promoter and E domains leads to stronger LPS responsiveness. This is consistent with the idea that the loop configuration of the *junb* locus bringing about these two domains provides increased transcriptional reactivity.

## DISCUSSION

We have previously reported that *junb* is an immediate early gene in LPS-stimulated BMDCs and that its protein product, JunB, contributes to BMDC activation via stimulation of inflammatory cytokine genes (11). We have also shown that NF-κB plays a crucial role in fast and efficient *junb* transcriptional induction before collaborating with the JunB protein to induce genes, such as IL-6, IL-12 or TNFα, in these cells (11).

**Figure 9.**
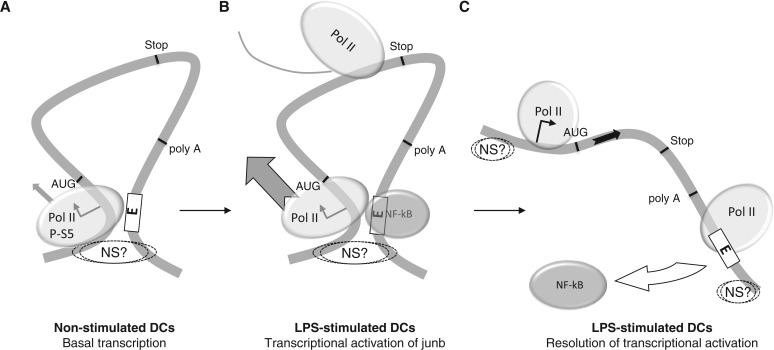
Model for transient transcriptional induction of *junb* in DCs. (**A**) Basal junb transcription in non-stimulated DCs*:* Pol II is largely paused by the TSS. The locus is organized in a short chromatin loop bringing the TSS and the E domain into close spatial proximity. This loop is anchored to an undefined intranuclear structure (NS?). (**B**) Transcriptional activation of junb. On LPS stimulation, NF-κB is activated and binds to the E domain. This fires Pol II activation, which is released on the gene body. (B) Resolution of junb transcriptional activation. NF-κB is released from E and the chromatin loop is relaxed. *junb* remains attached to a nuclear structure both upstream of the TSS and downstream of the E domain. Some paused Pol II is found in the TSS region and some Pol II having terminated transcription lags for some time in the E region.

As the mechanisms of *junb* induction remain poorly understood, we have investigated here how the downstream E enhancer domain of *junb*, which recruits NF-κB, could collaborate with the promoter region to activate transcription. To address this issue, we resorted to the mouse DC2.4 cell line where we first confirmed NF-κB- and E domain-dependent induction of *junb* in response to LPS (Figure 2). Then, we showed that, in non-induced DCs, the *junb* locus is organized in a short chromatin loop (<3–4 kb) that is most probably attached to a nuclear structure that remains to be defined. This brings the TSS and the E domains into close spatial proximity, which, most probably, provides a favorable topology for fast transcriptional activation upon binding of NF-κB to the E enhancer (see later in the text). Moreover, this loop is relaxed after induction. HRS- and 3C assay resolutions rely on the position of available restriction sites in the analyzed regions. Owing to this technical constraint, which is particularly limiting when dealing with short loci, we could unfortunately not establish whether the 2 NFRs identified by ChIP on each side of the *junb* gene (one including the TSS upstream region and the other the E domain) reside close to, or overlap partially with the two domains forming the stem of the chromatin loop. Whatever the answer to this issue, our data are consistent with the idea that most, if not all, major determinants of *junb* transcription control in DCs may reside within the chromatin loop (see model on Figure 9).

We also report that *junb* displays the essential characteristics of paused genes in non-stimulated DCs. Taken with data obtained in two other different cell contexts (26,27), this raised the possibility that Pol II pausing may be a ubiquitous characteristic of *junb* when silent or under condition of basal expression as in this study. Further systematic studies using other cell types are however required to establish this point firmly. The features of pausing we observed were high level of Pol II by the TSS coupled to CTD Ser5 phosphorylation, as well as the presence of NELF and DSIF in the same region. Moreover, transcriptional activation by LPS required the activity of CDK9 and was associated with loss of NELF, redistribution of DSIF on the *junb* locus and phosphorylation of CTD Ser2 to the detriment of that of CTD Ser5. Finally, we showed that active transcription-associated histone marks (H3K9Ac and H3K4Me3; as well as acetylation of H4, not shown) were already present on the *junb* gene body in non-stimulated DCs and increased on *junb* transcriptional activation. Interestingly also, an inactive transcription-associated histone mark, H3K9Me3, which persisted for at least 2 h post-stimulation (not shown), was observed on *junb* at the peak of transcriptional activity. Though paradoxical at first glance, this was consistent with the fact that *junb* gene activation by LPS is transient and suggested that both transcription activation and termination programs must be activated coordinately to avoid improperly high and/or protracted expression of this gene and, consequently, abnormal immune responses.

Our finding that the *junb* locus is organized in a chromatin loop in non-stimulated DCs raises a number of questions. At the present stage of investigations, we do not exclude that the reasons for, and the roles of, this loop configuration are multiple and non-exclusive. A first possibility would be that looping and attachment to a nuclear structure would insulate *junb* from its closest neighbor genes, PRDDX2 and HOOK2 (located ∼4 and 15 kb away, respectively), to avoid transcriptional cross-interferences and to ensure separate and differentiated expression, as has been suggested for other genes (65–67). Another possibility would reside in the recycling of Pol II during the period of basal *junb* transcriptional activity. In this scenario, transcription-terminating Pol II could easily be reloaded onto the TSS owing to spatial proximity with E to accomplish another round of *junb* transcription. It is possible that such a mechanism might concern other genes, as promoter–terminator interactions have already been observed in mammalian cells at loci such as BRCA1, CD68 or proviral HIV integrants, as well as in yeast (68–70). This mechanism would however no longer apply after LPS activation, as the chromatin loop is rapidly relaxed after transcriptional induction of *junb*. As the E region is also the transcriptional termination domain, a reasonable explanation for the detection of Pol II in the E region after *junb* has been activated would be an accumulation of slowed-down, transcription-terminating Pol II molecules, as has been observed in the transcription termination regions of various of other genes (71–73). A third possibility might be the enhancement of transcriptional directionality, as has recently been reported for certain yeast genes by Tan-Wong *et al.* (74) who postulated that looping would lead to directional histone deacetylation and subsequent repression upstream of the concerned promoters. This situation contrasts with that of many promoters (in both lower and higher eukaryotes) that have been shown to initiate bidirectional transcription, forming mRNAs on one side and short, labile, non-coding RNAs on the other (75). Though detailed investigation is required to assess whether the loop conformation of *junb* might orientate transcription towards the coding strand, the higher H3K9 acetylation levels we observed downstream of the TSS (Figure 3B) are consistent with Tai-Wong *et al.*’s hypothesis and the fact that we did not detect transcripts upstream of the TSS. The fourth and most straightforward possibility is that the spatial proximity of *junb* promoter and enhancer regions forms an environment particularly poised for transcription, explaining fast activation on NF-κB binding. This hypothesis is fully consistent with our observation that *junb* is a paused gene in non-stimulated DCs, as many such genes are particularly reactive for transcriptional activation on cell stimulation or stress.

The chromatin loop was found to be relaxed during *junb* transcriptional activity, most probably as a consequence of NF-κB binding, the latter protein being detected only in the E region (and not in the TSS domain) in ChIP experiments. Whether this relaxation participates actively to transcription termination by preventing Pol II recycling from the end of *junb* to the TSS constitutes an interesting possibility to explore. Another one to consider is, however, that fast reactivation of *junb* would simply be useless in terms of JunB protein accumulation after DCs have already been activated once. Supporting this idea, JunB protein, most probably due to stabilization mechanisms, continues to be present at high and stable levels till cell death occurring by 24–48 h post-LPS stimulation, i.e. long after *junb* mRNA levels have returned to basal level (11). It is of note that NELF-A (Figure 5A) in non-treated DCs does not show the bimodal distribution of Pol II and CDK9, which does not fit with the idea of a chromatin loop demonstrated by HRS- and 3C assays. At the present stage, we do not exclude an experimental bias linked to the ChIP assay protocol itself. Indeed, fixation of protein–DNA complexes by formaldehyde is only partial and requires delicate optimization to allow the immunoprecipitation steps, which possibly explains that certain events cannot be easily visualized.

Interestingly, several transcription factors have recently been shown to influence the establishment of local active chromatin conformation via induction of loops bringing about their cognate binding site (located upstream, within or downstream of activated genes and sometimes far away in intergenic regions) and the TSS domain in various genes. Such transcription factors include Klf1, GATA-1, -2 and -3, STAT6, ERα, AR, FXR, Lef1 as well as NF-κB [see (65–67,76)]. More specifically, NF-κB was shown to induce loop formation of the osteopontin (77) and iNos (78) genes in LPS-stimulated mouse macrophages, of the Igκ gene in LPS-stimulated mouse B lymphocytes (79) and of the peptidylarginine deiminase 1 gene in keratinocytes (80). All of these situations clearly depart from that of this work where the chromatin loop exists before the recruitment of NF-κB at the E enhancer domain, pointing to a mechanistical difference in the mode of action of this transcription factor depending on the gene and/or cell context [for more information, see (53)]. The process underlying transcription activation of *junb* by NF-κB in DCs is most probably multifactorial and complex. NF-κB/p65 having been shown to interact with CDK9 in other settings, it is tempting to speculate that an essential step in the release of blocked Pol II on the *junb* body might be contributed by NF-κB-dependent recruitment of CDK9, which would lead to CTD Ser2 phosphorylation as well as to phosphorylation-dependent elimination of NELF and conversion of DSIF from an inhibitor to an activator of transcription, as described elsewhere (29,60–62).

When the *junb* chromatin loop is formed during ontogeny and how it is maintained in DCs is still an open issue. Proteins such as CTCF, SATB1 and cohesin A have recently been shown to be instrumental for chromatin loop formation/maintenance and/or attachment to the nuclear matrix in specific studies [see (81–84)]. ChIP analyses of the −5000/+5000 region did not allow us to detect them on the *junb* locus in DCs (not shown). Moreover, although searches in the USCS genomic database showed histone distribution (including the 2 NFRs) on the *junb* locus similar to that in DCs, no such proteins could be found on, or close to, *junb* (not shown) in other cell contexts, suggesting that their implication in chromatin loops is not universal. As *junb* is not silent but subjected to basal transcription in non-stimulated DCs, it is important to consider that transcription factors/cofactors other than NF-κB might be key for chromatin loop configuration of the *junb* locus, as some of them have been shown to be essential for induction (see earlier in the text) or maintenance [see (65–67) for references] of chromatin loops at other loci. Along this line, it is of note that basal level of transcription, in unstimulated DCs, of the reporter plasmids used in the transfection experiments of Figures 2G, 8Ab and 8Bb are lower when all the κB-responsive sites are mutated. As binding sites for different transcription factors may overlap, it is possible that while mutating these κB sites, we have also affected the binding of transcription factors responsible for basal transcription of *junb* in non-stimulated DCs. Testing this, and the possibility that these putative factors are instrumental for chromatin loop formation and/or maintenance, will however have to await an extensive characterization of all transcription factors binding on either side of *junb*.

In conclusion, we report that, in non-stimulated DCs, *junb* is a paused gene and that its locus is organized in a short and stable chromatin loop bringing into close spatial proximity its upstream promoter regions and its downstream enhancer. Thereby, our work provides a novel topological framework to the transcriptional control of *junb*, which, most probably explains the fast response of this gene to an external stimulus such as LPS. The E enhancer domain contains, not only binding sites for NF-κB but also for other transcription factors that are crucial for rapid *junb* induction in other cell contexts/conditions (26,39–42). An important issue to solve in the future will therefore to establish whether activation of paused Pol II facilitated by chromatin loop configuration is a general trait of *junb* when the latter behaves as an immediate early gene induced by stimuli other than LPS and by transcription factors other than NF-κB.

## SUPPLEMENTARY DATA

Supplementary Data are available at NAR Online.

## FUNDING

MP’s laboratory is an ‘Equipe Labellisée’ of the French ‘Ligue Nationale contre le Cancer’; other fundings were from CNRS, the ARC and the ‘Programme Blanc’ of the Agence Nationale pour la Recherche (ANR); fellowships were from Ligue Nationale contre le Cancer (to T.G.) and Ministry of Higher Education and Research (MESR) and from the Ligue contre le Cancer (to F.C. and G.M.T.). Funding for open access charge: Laboratory funds.

*Conflict of interest statement*. None declared.

## Supplementary Material

Supplementary Data
