# Supplementary material for: Chromatin loop organization of the junb locus in mouse dendritic cells
Source: Nucleic Acids Res. 2013 Aug 5;41(19):8908–25. doi: 10.1093/nar/gkt669 (PMC3799436; doi:10.1093/nar/gkt669)
Supplement: Supplementary Data [file supp_41_19_8908__index.html]

Chromatin loop organization of the junb locus in mouse dendritic cells — Chromatin loop organization of the junb locus in mouse dendritic cells — Supplementary Data 

# Chromatin loop organization of the *junb* locus in mouse dendritic cells

## 

files

**Files in this Data Supplement:**

- Supplementary Data - pdf file
