# Supplementary material for: Chromatin loop organization of the junb locus in mouse dendritic cells
Source: Nucleic Acids Res. 2013 Aug 5;41(19):8908–25. doi: 10.1093/nar/gkt669 (PMC3799436; doi:10.1093/nar/gkt669)
Supplement: Supplementary Data [file supp_gkt669_nar-01568-x-2013-File011.pdf]

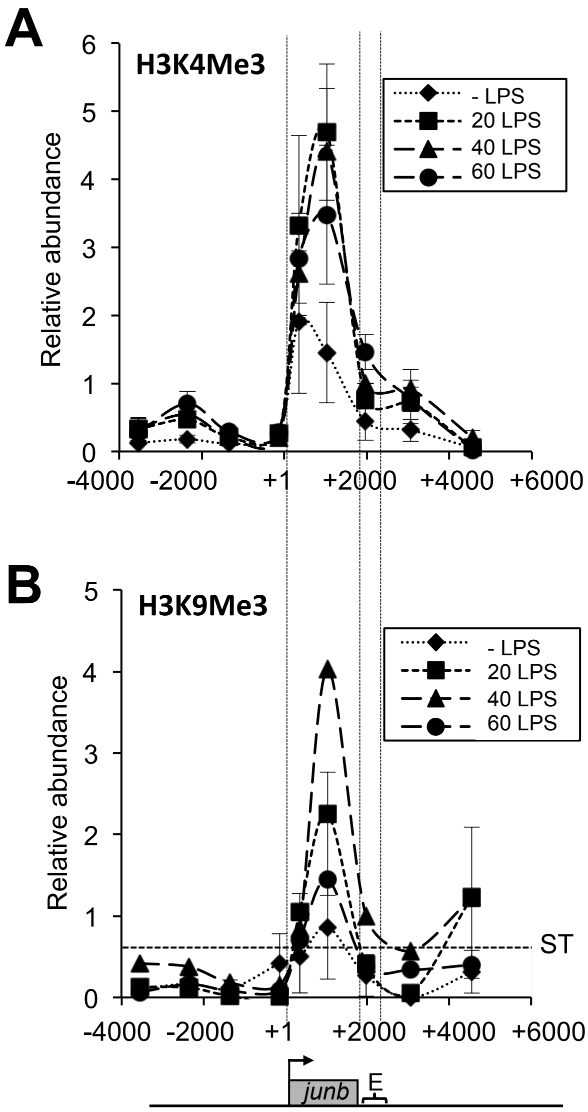

Supplementary Data 1: kinetic analysis of H3K4Me3 and H3K9Me3 modifications in LPS-stimulated DC2.4 cells. DC2.4 cells were stimulated by LPS as described in **Figure 3** and ChIP analyses were conducted at the indicated time points as in **Figures 3C and 3D**. The presented data are the average of 3 independent experiments ± SD.

Supplementary Table 1: PCR amplification oligonucleotides used for cloning of the *junb* DNA fragments used in Figure 8A and amplication of DNA fragments used in Figure 8B. The nucleotides in blue indicate the restriction sites used for cloning into the pGL3-Basic vector. The oligonucleotides indicated by (*) were also used to generate the DNA fragments that were PCR-amplified from the p-junb-Luc-κB- and the p-junb-Luc-κBmut reporter plasmids that were used in **Figure 8B**.

**Position in *junb***	**Sequences**	**Restric -tion site**	**Orien - tation**
Minimal promoter (*)	5'-GCTTGAggtaccCCTCCCCCTGCAGCCGCGCAGAG-3'	Acc65I	S
Minimal promoter	5'-GCTTGAggatccCTACGCTGGCCTGGCC-3'	BamHI	AS
wt Enhancer	5'-GCACTGCTCGAGCATGACCCCGCCCAGCAG-3'	Xho	S
wt Enhancer (*)	5'-GCTTGAcccGGGCCTGGGGCTTTCCGCGCCCAG-3'	SmaI	AS
Mut Enhancer (*)	5'-GCTTGAcccGGGCCTGGGGCGGCCGCGCCCAG-3'	SmaI	AS
wt Enhancer	5'-GCACTGggtaccCATGACCCCGCCCAGCAG-3'	Acc65I	S
wt Enhancer	5'-GCACTGggtaccCCTGGGGCTTTCCGCGCCCAG-3'	Acc65I	AS
Mut Enhancer	5'-GCACTGggtaccCCTGGGGCGGCCGCGCCCAG-3'	Acc65I	AS
